# Effect of Beech Sawdust Conditions Modification on the Efficiency of the Sorption of Anionic and Cationic Dyes

**DOI:** 10.3390/molecules29215017

**Published:** 2024-10-23

**Authors:** Paula Bugajska, Urszula Filipkowska, Tomasz Jóźwiak

**Affiliations:** Department of Environmental Engineering, University of Warmia and Mazury in Olsztyn, Warszawska St. 117a, 10-957 Olsztyn, Poland; paula.szymczyk@uwm.edu.pl

**Keywords:** beech sawdust, anionic dye, cationic dye, activation of the sawdust, epichlorohydrin, ammonia

## Abstract

The article presents studies on the effect of the modification method of beech sawdust on the sorption capacity of the anionic dye Reactive Black 5 (RB5) as well as the cationic dye Basic Violet 10 (BV10). In the studies, the pH value, the dose of sawdust activated with epichlorohydrin and the dose of ammonia were determined for the efficiency of the removal of anionic and cationic dyes. In the next phase of the study, the pH and the dose of epichlorohydrin activator on the previously activated sorbent were determined. The modification proposed in the work, which consists in the amination of sawdust in direct reaction with ammonia, increased the efficiency of the sorption of anionic dyes. This reaction increased the positive charge on the surface of the sorbent by introducing –NH_2_ groups, which led to an increase in the electrostatic attraction between the sorbent and the anionic dye, but to a decrease in the interaction between the sorbent and the cationic dye.

## 1. Introduction

Natural sorbents are considered to be materials with great potential, primarily due to their availability and low cost. These products are not always characterized by satisfactory dye removal capabilities, but due to their structure, they are considered to be materials that are highly susceptible to modification to achieve the desired properties. As a result of the modification, the physical parameters of the sorbent, such as the size of the specific surface area and the pore diameter, as well as the chemical parameters (e.g., addition of functional groups) can change. In many cases, it is necessary to pre-treat plant waste before using it as a sorbent. The treatment of naturally derived sorbents with different types of chemicals allows the extraction of soluble organic compounds that may interfere with the sorption of pollutants [1,2]. The pretreatment of sorbents using modifiers such as sodium and calcium hydroxide solutions, sodium carbonate, acid solutions (hydrochloric acid, nitric acid, sulfuric acid, tartaric acid, citric acid), organic compounds (ethylenediamine, formaldehyde, epichlorohydrin, methanol), oxidizing agents (hydrogen peroxide) to prepare them for the removal of organic compounds, dyes and heavy metals has been carried out by many researchers [3,4,5,6,7,8,9,10,11,12,13,14,15].

The treatment of lignocellulosic materials with sodium hydroxide can lead to a reduction in the degree of polymerization and crystallinity, disruption of the structural bonds between lignin and carbohydrates, disruption of the lignin structure and swelling of the material, resulting in an increase in surface area [16]. As a result of the reaction with the hydroxide, the porosity increases many times, which is accompanied by an increase in the volume and diameter of the pores [17]. According to the literature, the area and diameter of the pores of the sorbent can increase by a factor of 1.5–2 after the reaction with NaOH [5]. In addition, sodium hydroxide is the reagent that causes the conversion of the ester group to carboxylate and alcohol, as shown in the following equation [18]:

RCOOR + H_2_O _(in alkaline conditions)_ → RCOO^−^ + ROH (1)

The reaction with sodium hydroxide gives the surface of the sorbent a negative charge, increasing the electrostatic interaction between the adsorbent and the cationic adsorbate, which increases the efficiency of the sorption of pollutants such as heavy metals or cationic dyes [17,19,20,21]. According to the literature, the efficiency of the sorption of Zn^2+^ ions on pine sawdust reacting with sodium hydroxide can increase up to 15 times [22]. On the other hand, the efficiency of Cu^2+^ sorption can be increased more than 2.5-fold on sawdust treated with sodium hydroxide. The maximum sorption capacity for the unmodified sorbent was 5.43 mg/g and for the modified one was 13.95 mg/g [5].

Modification of lignocellulosic products with acids increases the porosity of the material. The SEM images also show that the outer surface of the sorbent is full of pores after the reaction with the acid, resulting in a larger specific surface area [23,24,25]. The EDX analysis shows that the treatment of natural sorbents with sulfuric acid leads to the formation of a material with a high sulfur content. This may be the result of sulfonation of lignocellulosic components leading to the formation of sulfonic functional groups on the surface of the material. In addition, the sorbents acquire a strongly acidic character. According to the pH_PZC_ literature, such sorbents can be between 2.7 and 3.3 in value [8,26]. The modification of natural products with acids increases the efficiency of the removal of heavy metals and cationic dyes [27,28,29,30]. The modification of lignocellulosic products with citric acid leads to the appearance of carboxyl groups in the structure of the sorbent, which are responsible for the sorption of impurities in the form of cations [31,32,33]. The reaction of cellulose products with succinic anhydride also contributes to the introduction of a carboxyl group into the structure of the sorbent [34].

The Lignocellulosic sorbents have hydroxyl groups in each monomeric unit of cellulose, lignin or hemicellulose so that they can react with carboxyl and amino groups of organic compounds [35,36]. Amino groups play an important role in the sorption of anionic pigments and heavy metals. Neutral polysaccharides acquire a basic character after the addition of an amino group to the polysaccharide chain, thanks to which they have the ability to electrostatically attract reactive and acidic dyes. Heavy metals, on the other hand, are removed from the amino group by the complexation of metal ions with a nitrogen atom. The addition of –NH_2_ groups to polysaccharides, therefore, also increases their sorption capacity towards heavy metals [37].

The enrichment of the sorbent with amino groups takes place through the amination process. Amination is a unitary chemical process that involves the introduction of an amino group –NH_2_ instead of a hydroxyl group –OH, a sulfonic group –SO_3_H, a ketone group =C=O, an aldehyde group –CHO, a halogen: Cl, Br and others. Popular aminating agents are m.in, melamine, diamine, ethylenediamine and triethylenetetramine. These are organic compounds from the amine group, which differ in the number of functional amino groups in the molecule [38].

Additional treatments are very often used to increase the efficiency of the amination process and the sorption of pollutants. These are usually reactions with activating factors, i.e., epichlorohydrin, formaldehyde. So far, the most common amination process has been carried out by the Mannich reaction, i.e., the amination of a sorbent in the presence of formaldehyde and a primary or secondary amine [39,40].

With this method of amination, however, it is difficult to control the reactive amino groups in the modified product, and the product contains more tertiary than primary and secondary amino groups. Tertiary groups in turn limit the applicability of aminated products [41]. Using the Mannich reaction to introduce amino groups into the structure of lignin used for the sorption of lead enabled a more than four-fold increase in sorption capacity compared to unmodified lignin [42]. The most commonly used activation factor is epichlorohydrin. This is an organic chemical compound from the epoxide group with two functional groups—epoxide and chloride substituent. Depending on the reaction of the activation process, an epoxy group or a chloride substituent is attached to the hydroxyl group of the polysaccharides, which are more reactive and enable more efficient product amination. According to the literature, the amination of natural products with initial activation enables a multiple increase in sorption capacity towards heavy metals and anionic dyes. The sorption capacity of unmodified peanut shells towards Sunset Yellow dye was 27.2 mg/g, while the shells pre-activated with epichlorohydrin were 4.3 times higher at 117.6 mg/g [43]. On the other hand, the removal efficiency of the anionic pigment Reactive Brilliant Red K-2BP by walnut shells aminated with epichlorohydrin-activated triethylenetetramine was up to 10 times higher than that of the unmodified sorbent [7].

The amination of organic compounds can also be carried out by an ammonia reaction, i.e., by a reaction with ammonia, which is many times cheaper. According to the literature, however, this reaction must take place at a very high pressure, elevated temperature and an excess of the aminating agent [44]. This process can also be carried out under normal conditions (1 a.m., 25 °C) if the sorbent is pre-activated by reaction with epichlorohydrin.

In addition to the type of innovating and activating substance, the dosage of the modifying substances and the conditions of modification are very important factors that influence the efficiency of the amination process, and thus, the achieved sorption capacity [41]. This type of research is rare in the literature. Depending on the activation conditions, there are two ways in which the reactivity of the sorbent can be affected. At an acidic pH (pH 2–4), epichlorohydrin condenses with a polysaccharide and the epoxy group is involved in the process. During the reaction, the epoxy ring is broken and the sorbent acquires chloride substituents. Since the polysaccharide has chloride groups, it can easily undergo the amination reaction. As a result of the alkylation, the amination agent attaches itself to the structure of the sorbent and enriches it with amino groups. In an alkaline environment (pH 10–13), however, the epichlorohydrin reacts with the polysaccharide in an alkylation reaction. As a result of the reaction, the polysaccharide acquires epoxy groups derived from the epichlorohydrin. During amination, the epoxy groups undergo a condensation reaction with the aminating agent. As a result, the sorbent receives additional amino groups [45]. The dose of activating and oiling factors also has a considerable influence on the effectiveness of sorbents and their price. If too low of a dose of modifiers is used, the sorbent will not achieve satisfactory efficiency in removing impurities from aqueous solutions. On the other hand, an excessive dose of aminating and activating agents significantly increases the cost of producing the sorbent.

Polysaccharides are components of many waste products from the agricultural and food industries. Although these products do not reach the sorption capacity of activated carbon, more and more attempts are being made to use them as sorbents due to their low price. The prospect of increasing the sorption capacity of available and cheap sorbents by aminating the polysaccharides they contain can significantly reduce the cost of industrial wastewater treatment.

The efficiency of amination, i.e., the number of amino functional groups introduced into the polysaccharide, depends primarily on the process parameters. The most important of these are the dose of the aminating agent and the substances that support the amination process (activating substances), as well as the conditions of modification, i.e., the pH value.

The article presents a method for the amination of beech sawdust with a 25% aqueous ammonia solution with an initial activation of epichlorohydrin. For the sorbents obtained, the effectiveness of the binding of dyes commonly used in the textile industry in the sorption process was tested. The work contributes to the knowledge about the possibility of modifying products of natural origin and the influence of the conditions and method of modification on the efficiency of amination.

The research results show that properly prepared sorbents of natural origin can be a cheaper and more effective alternative to commonly used sorbents. The possibility of enriching the polysaccharides contained in sawdust with amino groups and thus increasing sorption efficiency has great potential.

The research presented in the work consisted of finding the best conditions for preparing a sorbent of natural origin, such as sawdust, such as the dose and pH of activation with epichlorohydrin and the pH and dose of activation with ammonia. The value of the research also lies in showing how important it is to combine the activation conditions of epichlorohydrin and ammonia.

## 2. Results and Discussion

### 2.1. Effect of pH of Sawdust Activation with Epichlorohydrin on the Efficiency of Dye Sorption

The efficiency of the sorption is largely influenced by the structure of the sorbent. However, the sorption efficiency of modified sorbents depends on the type of modifier used and the conditions of the modification process. These factors are not universal and depend on both the sorbent and the sorbate.

An anionic dye RB5 and a cationic dye BV10 were selected for the study on the influence of pH on the efficiency of modification, the type of modifier and its dose. Both dyes are frequently used in sorption research, which makes it easier to compare the results with those of other authors.

**Figure 1:**
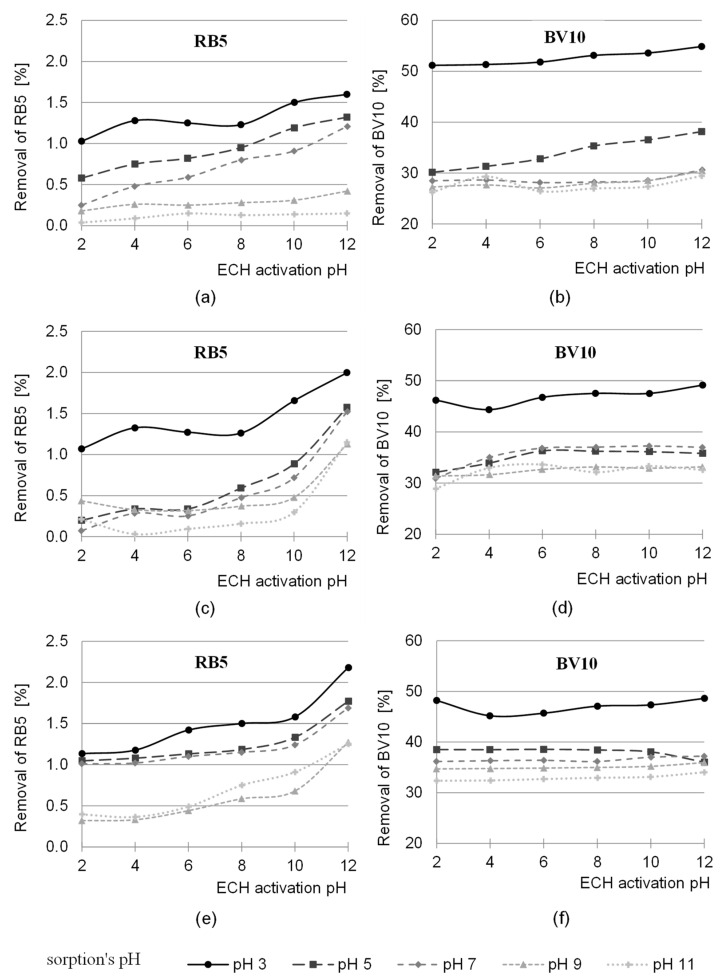
The effect of pH activation of sawdust with epichlorochydrin (ECH) on the sorption efficiency of RB5 and BV10 dyes at different pH values. Ratio of the functional groups of ECH to the hydroxyl groups of the sawdust: (**a**,**b**) 1:3; (**c**,**d**) 1:1; (**e**,**f**) 3:1.

The pH range of the activation process tested in the work was between pH values of 2.0 and 12.0. The study was conducted for three ratios of the functional groups of the activating agent to the hydroxyl groups of the sawdust—1:3, 1:1 and 3:1. The effect of pH on the efficiency of the modification of sawdust with epichlorohydrin is shown in Figure 1.

The highest removal efficiency of anionic and cationic dyes was achieved for the sorbent activated with epichlorohydrin (ECH) at pH 12 (Figure 1). In general, the sorption efficiency of both dyes on S-AB-E (sawdust activated with epichlorohydrin) increased with the increase in pH of the modification of this sorbent. The greatest influence of the pH value of the sorbent activation on the sorption capacity was found in the test series with RB5. The sorption efficiency of this dye on S-AB-E modified at pH 12 was even several times higher compared to the same sorbent modified at pH 2 (Figure 1c, series with pH 7).

Such a relationship was observed for all three ratios of the epichlorohydrin functional groups to the hydroxyl groups of the polysaccharides.

### 2.2. Effect of Epichlorohydrin Dose on Sorption Capacity of Sorbent

**Figure 2:**
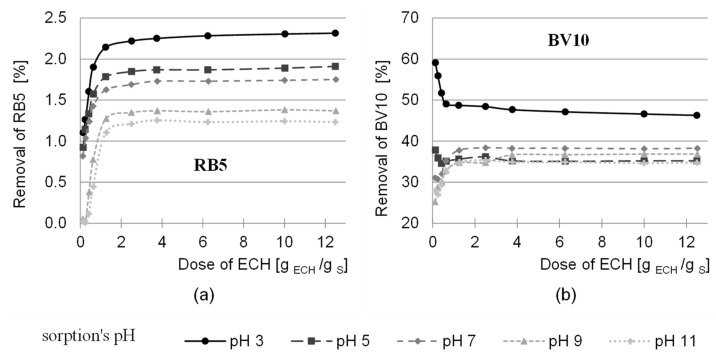
Effect of ECH dose during sawdust activation on the sorption efficiency of (**a**) RB5, (**b**) BV10 on the tested sorbent at different pH values. Sorption time: 2 h, temperature 25 °C.

The sorption efficiency of the modified sorbent was tested with the anionic reactive dye, Reactive Black 5 (RB5), and the cationic dye Basic Violet 10 (BV10). The sorption efficiency of the sorbent, depending on the dose of the activation factor used, is shown in Figure 2.

The ability to remove anionic pigment by sawdust activated at pH 12 increased with increasing dose of epichlorohydrin used, up to the dose of activation factor 6.25 g ECH/g sorbent (ratio 5:1). A further increase in the dose had no significant effect on the efficiency of the sorption process. The higher the dose of epichlorohydrin in the activation reaction at pH 12 in the sorbent, the more epoxy groups can react with the reactive groups (vinyl sulfone) of the RB5 dye. As a result, the anionic dye is removed from the solution by chemisorption. On the other hand, above the epichlorohydrin dose of 6.25 g/g sorbent (5:1 ratio), the availability of free –OH groups for epoxy groups are limited, so that the amount of bound dye is not increased. The highest dye removal efficiency was achieved at a sorption pH of 3.

The increasing efficiency of the sorption of Reactive Black 5 in the pH range of 11 < 9 < 7 < 5 < 3 results from the increasing protonation of the hydroxyl groups of the polysaccharides present in the sawdust. Lowering the pH value of the sorption led to an increasing positive charge of the sorbent, resulting from a larger number of –OH^2+^ groups, which led to a stronger interaction with the negatively charged dye [46].

In the case of the cationic dye, the highest dye removal efficiency was also achieved at pH 3, but the sorption efficiency decreased as the dose of epichlorohydrin was increased.

The decrease in the sorption efficiency of BV10 at a sorption pH below 7 with an increase in the dose of epichlorohydrin could be due to the presence of a carboxyl group in the dye structure. In a solution below pH 7, some hydroxyl groups are protonated, which gives the sorbent a positive character [47]. The protonation of the hydroxyl groups of the sorbent causes an increase in the electrostatic interaction with the negatively charged carboxyl groups of the BV10 dye. Epichlorohydrin, on the other hand, reacts with the –OH groups of the polysaccharides, so that the availability of the protonated hydroxyl groups of the polysaccharide decreases with increasing doses of the activating factor and the efficiency of the sorption of the BV10 dye decreases [48].

At a pH ≥ 7, an opposite trend was observed in the efficiency of the sorption of cationic pigments with an increase in the dose of epichlorohydrin. At a solution pH above 7, the hydroxyl groups of the sorbent do not protonate, so the electrostatic interaction between the dyes and the sorbent is less important. In the pH range of 7–11, hydrogen bonds and chemisorption play a greater role in the sorption process. Epoxy groups formed by the reaction of the sawdust with epichlorohydrin can permanently bind the BV10 dye by reacting with the -COOH group. This could explain the increase in the efficiency of the sorption of the cationic dye at neutral and alkaline pH with increasing epichlorohydrin dose [49].

### 2.3. Effect of Ammonia Dose on the Efficiency of Dye Removal

**Figure 3:**
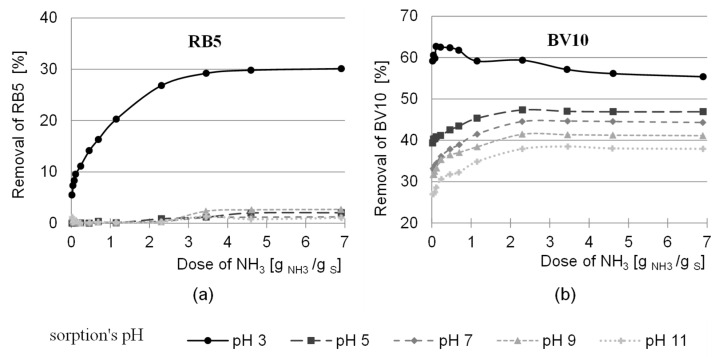
Effect of ammonia dose during sawdust modification on the sorption efficiency of (**a**) RB5, (**b**) BV10 on the tested sorbent at different pH values.

The sorption capacity of the aminated sawdust was tested with the anionic dye, Reactive Black 5 (RB5), and the cationic dye, Basic Violet 10 (BV10). The sorption capacity of the sorbent, depending on the dose of aminating agent used, is shown in Figure 3.

The RB5 removal capacity of S-AB-A increased with increasing the dose of ammonia used, up to a dose of 3.45 g NH_3_/g sorbent (ratio 15:1). A further increase in the dose had no significant effect on the efficiency of the sorption process. The highest efficiency of dye removal was achieved at pH 3. After 2 h of sorption, the dye removal efficiency at pH 3 increased by about 25% compared to the sorbent where the ammonia dose was 0.0023 g NH_3_/g sorbent.

The increase in the efficiency of the sorption of anionic dyes pigment sorption with the increase in ammonia dose was associated with an increase in the number of amino groups in the sorption structure. According to the literature, it is the amino groups that are responsible for the sorption of anionic dyes, due to their ability to protonate, and thus, the strong electrostatic interaction between the anionic groups of the sorbent and the dye [50]. On the other hand, the increasing efficiency of the sorption of Reactive Black 5 (RB5) in the pH range 11 < 9 < 7 < 5 < 3 is due to the increasing number of protonated amino groups in the chemical structure of the polysaccharides. Lowering the pH of the solution led to an increasing positive charge of the sorbent, which in turn led to a stronger interaction with the negatively charged dye [46].

In the case of the cationic dye BV10, the highest dye removal efficiency was also achieved at pH 3, but this efficiency decreased with increasing ammonia dose. The decrease in sorption efficiency with an increase in the ammonia dose is related to a decrease in the amount of available hydroxyl groups of the polysaccharides responsible for the sorption of the BV10 dye. In the other test series, an opposite trend was observed at a sorption pH above 3, i.e., the efficiency of BV10 dye sorption increased as the ammonia dose was increased. At higher pH values, the electrostatic interaction between the sorbent and the sorbate is less important, while other forces such as the van der Waals interaction and hydrogen bonding play a greater role. After amination, the sorbent is able to form more hydrogen bonds with the dye than the unmodified sorbent. Therefore, the moreNH_2_ groups that are added, the greater the efficiency of BV10 dye removal [47,51].

### 2.4. Effect of pH of Activation with Epichlorohydrin on the Sorption Efficiency of Aminated Sawdust

**Figure 4:**
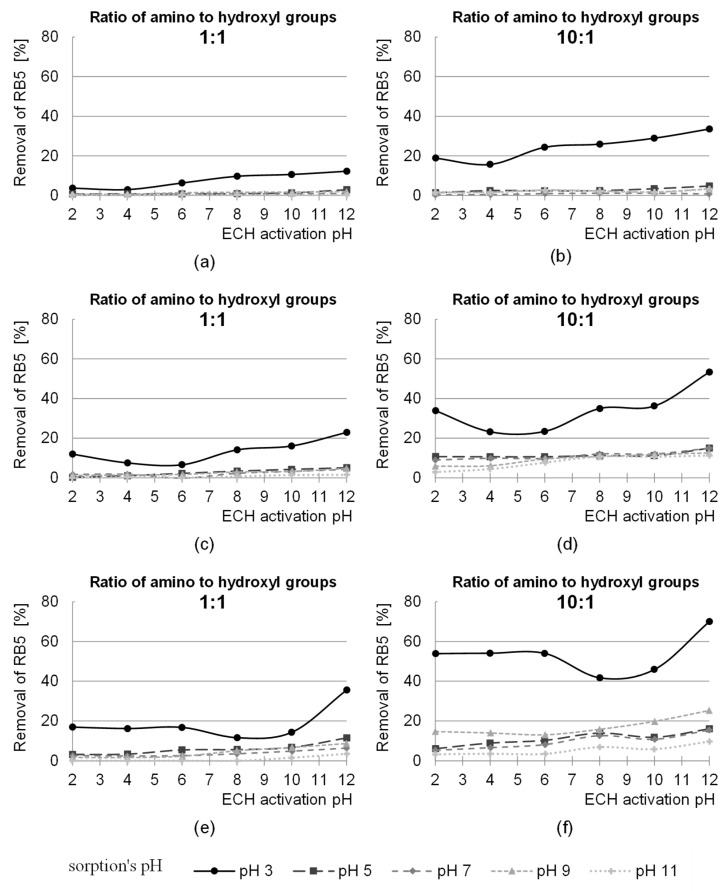
Effect of pH activation of ECH sawdust (sawdust was subsequently aminated) on the sorption efficiency of RB5 dye on the tested sorbent. Ratio of ECH functional groups to sawdust hydroxyl groups during their activation: (**a**,**b**) 1:3; (**c**,**d**) 1:1; (**e**,**f**) 3:1. Ratio of amine functional groups to sawdust hydroxyl groups during their amination: (**a**,**c**,**e**) 1:1; (**b**,**d**,**f**) 10:1.

**Figure 5:**
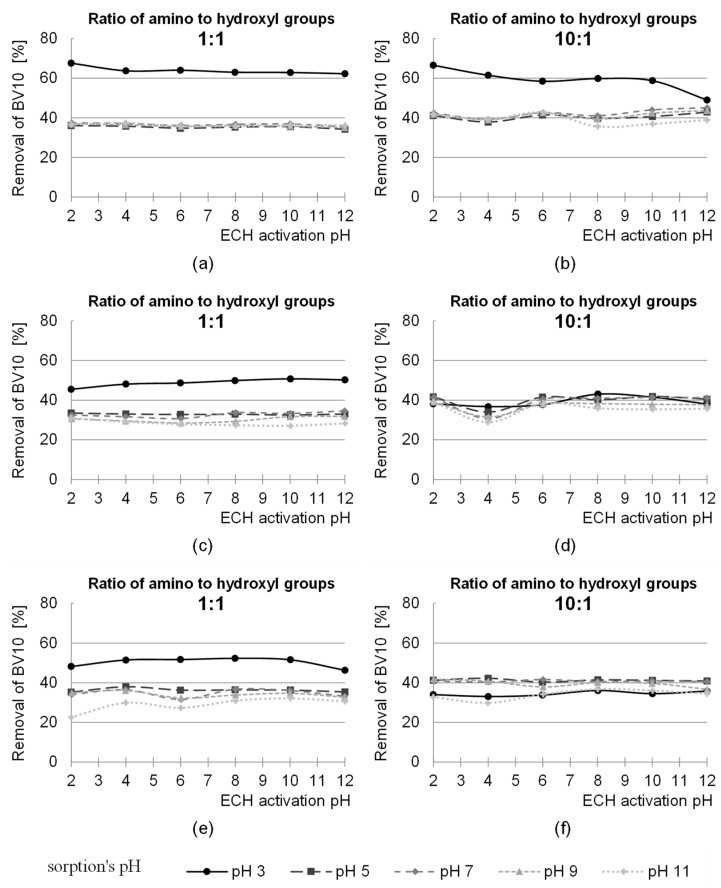
Effect of pH activation of ECH sawdust (sawdust was subsequently aminated) on the sorption efficiency of the BV10 dye on the tested sorbent. Ratio of the functional groups of ECH to the hydroxyl groups of the sawdust during their activation: (**a**,**b**) 1:3; (**c**,**d**) 1:1; (**e**,**f**) 3:1. Ratio of the amine functional groups to the hydroxyl groups of the sawdust during their amination: (**a**,**c**,**e**) 1:1; (**b**,**d**,**f**) 10:1.

The pH range of the activation tested in the work was between 2.0 and 12.0 pH. The study was carried out for three ratios of the functional groups of the activating agent to the hydroxyl groups of the sawdust—1:3, 1:1 and 3:1 (doses of 0.417, 1.250 and 3.750 g ECH/g sorbent respectively) and two ratios of the amino groups to the hydroxyl groups 1:1 and 10:1 (doses of 0.23 and 2.3 g NH_3_- H_2_O/g sorbent). After 2 h of sorption, the effectiveness of the sorption of anionic (RB5) and cationic (BV10) pigments was analyzed at pH 3.0; 5.0; 7.0; 9.0 and 11.0. The effects of sawdust activation pH on the efficiency of dye sorption on the amination-treated sorbent are shown in Figure 4 and Figure 5.

In most test series, the sorption efficiency of the RB5 dye on modified sawdust increased with an increase in sorbent activation pH, with the highest efficiency being achieved at an activation pH of 12 regardless of the epichlorohydrin dose (Figure 4). The need to create an alkaline environment for the reaction with epichlorohydrin, which then enables effective amination, is also confirmed by the literature data [52,53]. According to the literature, epichlorohydrin undergoes an alkylation reaction with the hydroxyl groups of the polysaccharide in an alkaline environment. As a result of the reaction, the polysaccharide acquires epoxy groups, which are derived from the epichlorohydrin and enable the reaction with the aminating agent. As a result, the sorbent acquires additional amino groups [45].

The efficiency of anionic pigment removal at an activation pH of 12 increased with the dose of epichlorohydrin (1:3, 1:1, 3:1) and ammonia (1:1, 10:1) (Figure 4). The highest RB5 dye removal efficiency was achieved at a sorption pH of 3. The increase in dye removal efficiency for the 1:1 ammonia ratio and the 1:3, 1:1 and 3:1 epichlorohydrin ratios was 10%, 16% and 25%, respectively. However, for an ammonia ratio of 10:1 and an epichlorohydrin ratio of 1:3, 1:1 and 3:1, the removal efficiencies were 18%, 27% and 28%, respectively (Figure 4). The increased removal efficiency of Reactive Black 5 (RB5) with an increase in the dose of epichlorohydrin and ammonia is due to an increase in the number of amino groups responsible for anion sorption.

In the case of the cationic dye, the pH value of the activation had no clear influence on the sorption efficiency (Figure 5). In most of the test series, the dose of epichlorohydrin and ammonia was also of little significance. Only at a sorption pH value of 3 was a decrease in the sorption efficiency of the BV10 dye with an increase in the activation pH value observed at higher doses of both modifiers. Under these conditions, the number of bound amino groups was highest, increasing the alkaline character of the sorbent. The positive charge on the surface of the sorbent, resulting from the presence of a large number of protonated amino groups, makes it difficult for the cationic dye BV10 to reach the sorption center. In addition, the hydroxyl groups of the polysaccharides are involved in the reaction with epichlorohydrin, which reduces the number of –OH groups available for the cationic dye. This is indirect evidence for the occurrence of amination, since at the time of the introduction of amino groups, the sorbent acquires a positive charge and thus the electrostatic attraction between the sorbate and the sorbent decreases.

### 2.5. Effect of Epichlorohydrin Dose on the Efficiency of Dye Sorption on an Amination-Treated Sorbent

**Figure 6:**
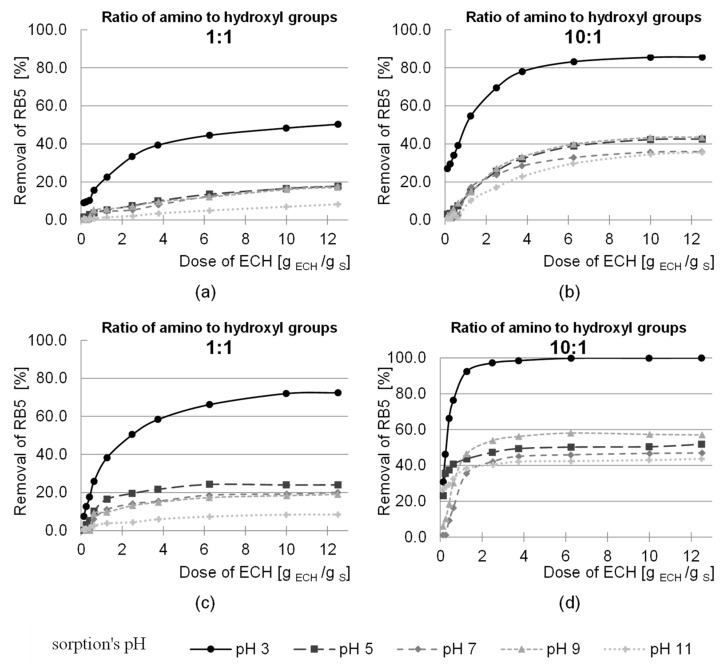
Effect of ECH dose during sawdust activation (which preceded amination) on the efficiency of RB5 sorption on modified sawdust. Ratio of amine functional groups to hydroxyl groups of sawdust during their amination: (**a**,**c**), 1:1; (**b**,**d**) 10:1. Sorption time: (**a**,**b**) 2 h; (**c**,**d**) 24 h.

**Figure 7:**
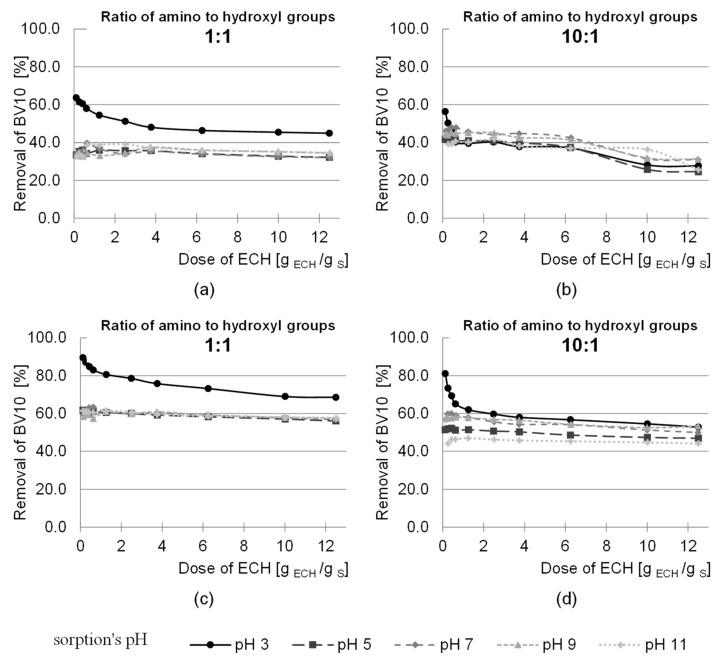
Effect of ECH dose during sawdust activation (preceding amination) on the sorption efficiency of BV10 on modified sawdust. Ratio of amine functional groups to hydroxyl groups of sawdust during their amination: (**a**,**c**), 1:1; (**b**,**d**) 10:1. Sorption time: (**a**,**b**) 2 h; (**c**,**d**) 24 h.

The study was carried out for the ratios of the functional groups of the activating agent to the hydroxyl groups of the sawdust from 1:10 to 10:1 (dose 0.125 to 12.5 g ECH/g sorbent) and two ratios of the amino groups to the hydroxyl groups 1:1 and 10:1 (dose 0.23 and 2.3 g NH_3_- H_2_O/g sorbent). After 2 and 24 h of sorption, studies were carried out on the sorption efficiency of anionic (RB5) and cationic (BV10) dyes at pH 3.0; 5.0; 7.0; 9.0 and 11.0. The effect of epichlorohydrin dose on the dye sorption efficiency on the amination treated sorbent is shown in Figure 6 and Figure 7.

The efficiency of the removal of anionic pigment increased with the dose of epichlorohydrin (1:10–10:1) and ammonia (1:1, 10:1) (Figure 6). The pre-activation of products of natural origin enables a higher efficiency of amination. According to the literature, better amination effects are achieved when the sorbent has more reactive epoxy groups instead of hydroxyl groups, which makes it easier to bind amino groups. On the other hand, the more amino groups capable of protonation, the better the sorption efficiency of anionic dyes [54,55].

Sorption of the RB5 dye was most effective at pH 3. The increase in dye removal efficiency at this pH sorption after 2 h of the process for an epichlorohydrin dose of 12.5 g ECH/g sorbent and an ammonia ratio of 1:1 (dose 0.23 g NH_3_- H_2_O/g sorbent) was about 41% compared to the lowest dose of epichlorohydrin used in the study (Figure 6). On the other hand, when comparing the sorption efficiency at the highest dose of epichlorohydrin with the sorption efficiency at the lowest dose of epichlorohydrin for an ammonia ratio of 10:1 (dose 2.3 g NH_3_- H_2_O/g sorbent), the increase in RB5 sorption efficiency was 58%. After 24 h of sorption at an epichlorohydrin dose of 12.5 g ECH/g sorbent and an ammonia ratio of 1:1, the increase in dye removal efficiency was 65% compared to an epichlorohydrin dose of 0.125 g ECH/g sorbent (Figure 6). In comparison between the highest and lowest epichlorohydrin dose for an ammonia ratio of 10:1, the increase in RB5 sorption efficiency was 70%. After 2 h of sorption, an increase in dye removal efficiency was observed for an epichlorohydrin dose of 12.5 g ECH/g sorbent, but after 24 h, a dye removal efficiency of 100% was already achieved at a dose of 6.25 g ECH/g sorbent (5:1 ratio). A further increase in the dose had no effect on the sorption process; therefore, a ratio of epichlorohydrin to the hydroxyl groups of the sawdust of 5:1 was used in the subsequent studies.

In the case of the cationic dye BV10, the opposite trend was observed with respect to the sorption of the anionic dye RB5. The lowest sorption efficiency was obtained for the highest dose of epichlorohydrin (Figure 7). The hydroxyl groups of the polysaccharides are responsible for the sorption of the BV10 dye, while with increasing epichlorohydrin dose, the number of –OH groups available for the cationic dye decreases, thus decreasing the sorption efficiency. Due to the fact that the sorption efficiency of the cationic dye decreases with the introduction of additional functional groups from epichlorohydrin or ammonia into the structure of the polysaccharides, further studies on the modification of sawdust were carried out only for the anionic dye.

### 2.6. Determining the Dose of Ammonia After Epichlorohydrin Activation

**Figure 8:**
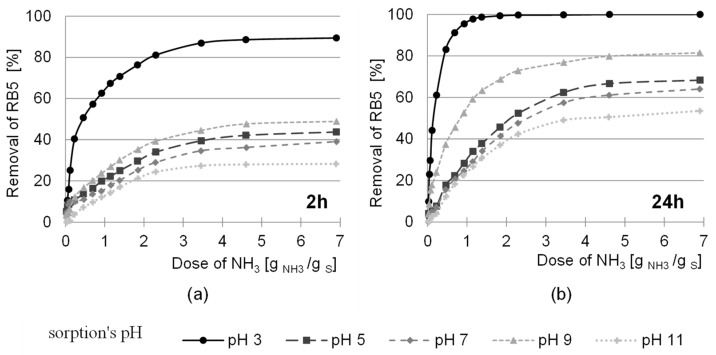
Effect of the ammonia dose after the initial activation of sawdust from ECH with a dose of 6.25 g ECH/g S-AB on the sorption efficiency of the anionic pigment RB5 after (**a**) 2 h, (**b**) 24 h sorption.

The studies were carried out for the ratio of amino groups to hydroxyl groups of the sawdust from 1:10 to 30:1 (dose of 0.23 to 6.9 g NH_3_- H_2_O/g sorbent). After 2 and 24 h of sorption, the removal efficiency of the anionic pigment (RB5) was determined at pH 3.0; 5.0; 7.0; 9.0 and 11.0. The effect of the amination dose after the initial epichlorohydrin activation with a dose of 6.25 g ECH/g sorbent (5:1 ratio)—selected based on previous studies—on the sorption efficiency of RB5 is shown in Figure 8.

The removal efficiency of the anionic dye increased with the ammonia dose from 0.023 to 6.90 g of 25% aqueous ammonia solution per 1 g d.m. S-AB (ratio 1:10–30:1). Sorption of the RB5 dye was most effective at pH 3. The increase in dye removal efficiency at this pH for the ammonia ratio of 30:1 compared to the lowest ratio of 1:10 was 85% after 2 h of sorption and 90% after 24 h (Figure 8). The increase in the efficiency of the sorption of anionic dyes is due to the fact that as the ammonia dose is increased, the number of bound –NH_2_ groups responsible for the sorption of RB5 increases. After 2 h of sorption, an increase in dye removal efficiency was observed for an ammonia dose of 6.90 g NH_3_/g S-AB, but after 24 h, a dye removal efficiency of 100% was already achieved at a dose of 2.30 g NH_3_/g S-AB (ratio 10:1). A further increase in the dose had no effect on the sorption process; therefore, the ratio of amino groups to hydroxyl groups of the polysaccharide 10:1 (dose 2.30 g NH_3_/g S-AB) was used in the subsequent studies. The increase in the amount of anionic pigment removed after 24 h of sorption was related to the loosening of the sorption structure, which gave the dye access to the sorption centers in the deeper layers of the sorbent.

### 2.7. FTIR Analysis

**Figure 9:**
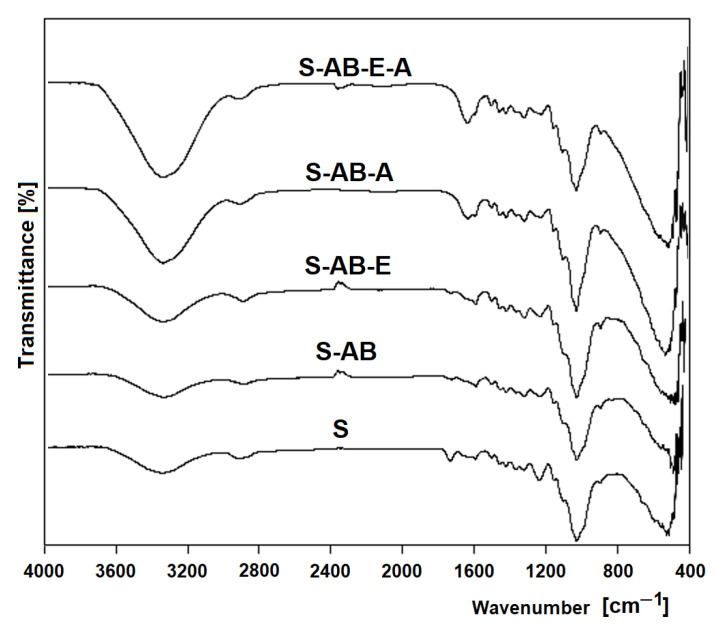
FT-IR spectra of the sorbents tested.

The FTIR analysis was carried out for all cellulose sorbents tested (Figure 9).

In all spectra, there is a clear peak between 3600 and 3300 cm^−1^, which is associated with the vibrations of the OH ring—and of the side chains (CH-OH) and (CH_2_-OH) [56]. Absorption in the area of 3000–2800 cm^−1^ is assigned to -CH groups [57,58]. The peak in the area of 1743 cm^−1^ responsible for the C=O vibrations in the spectrum of the sawdust treated with H_2_SO_4_ and NaOH (S-AB) is much smaller than in the spectrum of the unmodified sawdust (S), while it is absent in the following spectra. A decrease or disappearance of the peak may indicate a C=O reaction with modifying factors [54,59]. In all spectra, there are several bands in the range of 1500–1200 cm^−1^ that correspond to the deformations of the primary and secondary –OH groups, and between 1200 and 1000 cm^−1^, the stretching bands of the -CO groups are visible [58]. In the spectra of sawdust reacted directly with ammonia (S-AB-A) and in the spectrum of sawdust aminated after initial activation (S-AB-E-A), a characteristic spectrum of 1640–1660 cm^−1^ appears, which is responsible for the deformation of the amino group [58,60,61]. This peak is evidence of the amination process in which amino groups are introduced into the structure of polysaccharides.

## 3. Materials

### 3.1. Beech Sawdust

The beech sawdust used for the research, which was provided by the company “Kaczkan” (Małdyty, Poland), is a waste product from the production of wooden floors. An isolated fraction of sawdust with a particle size between 0.5 and 0.6 mm was used for the study. The moisture content of beech sawdust was 7%. The fiber fractions were determined using the Van Soest chemical analysis method [62]. This method consists of the selective separation of different fractions under specific conditions using surfactants. The characteristics of the raw material, expressed in % (*w*/*w*) in relation to the dry matter, are shown in Table 1.

### 3.2. Dyes

Anionic dye (Reactive Black 5) and cationic dye (Basic Violet 10), which are commonly used in the textile industry, were used for the study. The dyes came from the “Boruta” S.A (Zgierz, Poland). Dye Production Plant. The chemical structure and properties of the dyes are listed in Table 2.

### 3.3. Modifying Agents

In the study, epichlorohydrin >99% (analytically pure) from SIGMA—ALDRICH (Saint Louis, MO, USA) was used as an activating factor. A 25% aqueous ammonia solution of SIGMA—ALDRICH (Saint Louis, MO, USA) was used as an aminating agent in the work. The properties of epichlorohydrin and 25% aqueous ammonia solution are listed in Table 3.

### 3.4. Names of the Sorbents Used in the Tests

The names of the sorbents used in the tests are as follows:

S-AB—sawdust treated with acid and basic;
S-AB-E—sawdust treated with acid and basic and activated with epichlorohydrin;
S-AB-A—sawdust treated with acid and basic and activated with a 25% aqueous solution of ammonia;
S-AB-E-A—sawdust treated with acid and basic and activated with 25% aqueous ammonia solution, with initial activation of epichlorohydrin.

### 3.5. Chemical Reagents and Measuring Equipment

Sodium hydroxide in the form of microbeads (POCH S.A., Gliwice, Poland), hydrochloric acid 35–38% (POCH S.A., Poland), nitric acid 69% (POCH S.A., Poland) and pH 4.01/7.01/10.01 buffer solutions for pH meter calibration (Hanna Instruments, Olsztyn, Poland) were used in the study. All the reagents used in the tests were of the purity that was pure for analysis.

The following measuring equipment was used during the research—Genesys 20 spectrophotometer (Thermo Scientific, Waltham, MA, USA), HI 221 pH meter (Hanna Instruments, Olsztyn, Poland) and ALPHA FT-IR-base spectrometer (Bruker, Karlsruhe, Germany), ANKOM 220 fiber analyzer (ANKOM Technology, Macedon, NY, USA).

## 4. Methodology

**Figure 10:**
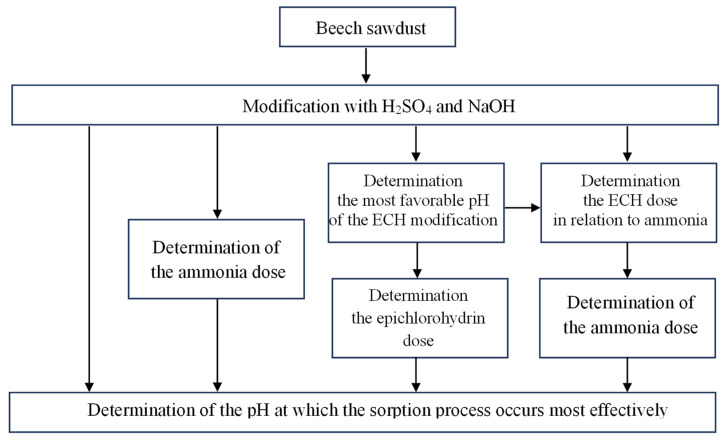
Study design.

The diagram of the organization of the research is presented below (Figure 10).

### 4.1. Preparation of Dye Solutions

To prepare a basic solution with a concentration of 10,000 mg/dm^3^, 10 g of dye was weighed into a beaker (0.05 dm^3^) on an analytical balance, then the dye was transferred to a volumetric flask (1 dm^3^) and made up to a volume of 1 dm^3^ with distilled water. The dye solution prepared in this way was stored in a laboratory refrigerator at 4 °C. Working solutions of dyes were prepared from the stock solutions in the following concentrations: 1, 2, 5, 10, 25, 50, 75, 100, 150, 200, 400, 600, 80, 1000, 1500, 2000, 3000 mg/dm^3^ (for anionic dye RB5) and 10, 25, 50, 75, 100, 200, 250, 300, 500 mg/dm^3^ (for cationic dye BV10).

### 4.2. Preparation of Epichlorohydrin and Ammonia Solutions

The basic solutions of the activating and aminating agent were prepared using the weighing method. The required amount of the compound (depending on the experiment) was weighed into beakers with a capacity of 1 dm^3^ and made up to 1000 g with distilled water. Thus, the solutions prepared solutions were stored in a laboratory refrigerator at a temperature of 4 °C. The correction of the pH value with NaOH and HCl solutions was carried out for the working solutions of epichlorohydrin immediately before the reaction with the sorbent.

### 4.3. Treating Sawdust with Sulfuric Acid and Sodium Hydroxide (S-AB)

To remove mineral substances, the sawdust was washed with 2 M sulfuric acid for 24 h. Then, the beech sawdust was washed with 1 M sodium hydroxide for 24 h to remove the proteins and loosen the structure of the sorbent. After each reaction, the sawdust was washed with distilled water until the pH of the solution after washing the sorbent was 7.0 ± 0.1. The beech sawdust was then dried at 100 °C for 48 h.

### 4.4. Preparation of an Epichlorohydrin-Activated Sorbent (S-AB-E)

The procedure for activating the sorbent consisted of a bath in appropriately prepared working solutions of epichlorohydrin. After reaction with sulfuric acid and sodium hydroxide (S-AB), 2 g of beech sawdust were weighed into Erlenmeyer flasks with a capacity of 200 cm^3^ and then mixed with epichlorohydrin solutions with a specific pH and a volume of 100 cm^3^. The flasks were then placed on a water bath at a temperature of 60 °C. After 24 h, the modified sawdust (S-AB-E) was washed in a laboratory sieve with distilled water to wash out the unreacted medium. The sorbent prepared in this way was stored in distilled water in a laboratory refrigerator at a temperature of 4 °C.

### 4.5. Preparation of the Aminated Sorbent (S-AB-A)

The amination procedure consisted of bathing the sorbent in appropriately prepared ammonia solutions. The 2 g of beech sawdust was weighed into Erlenmeyer flasks with a capacity of 200 cm^3^ after a reaction with sulfuric acid and sodium hydroxide (S-AB) and then ammonia solutions with a pH of 12 were added in a volume of 100 cm^3^. The flasks were then placed on a shaker (150 rpm) for 24 h. The amination reaction was carried out at room temperature (25 ± 1 °C). After 24 h, the modified sawdust (S-AB-A) was washed in a laboratory sieve with distilled water to wash out the unreacted medium. The sorbent prepared in this way was stored in distilled water in a laboratory refrigerator at a temperature of 4 °C.

### 4.6. Preparation of an Aminated Sorbent with Epichlorohydrin Preactivation (S-AB-E-A)

The activated sorbent was prepared according to the methodology presented in Section 4.4. The sorbent was then subjected to an amine reaction while rinsing out the unreacted epichlorohydrin according to the methodology in Section 4.5. The sorbent prepared in this way was stored in distilled water in a laboratory refrigerator at a temperature of 4 °C.

### 4.7. FTIR Analysis

The functional groups on the sorbent surface and the type of bonds present in the sorbents were determined in the infrared range using a Fourier transform spectroscope, the total internal reflection method with an ATR attachment with single reflection. The lignocellulosic sorbents were dehydrated in a hydraulic press before being analyzed. A suitably prepared test was pressed against the crystal with a specific force. After each measurement, the diamond crystal of the ATR attachment was washed with acetone (CH_3_COCH_3_), then the background was scanned, and a new series was placed on the crystal. The FTIR spectra were recorded in the specific infrared range (wavelength 4000–600 cm^−1^) with a resolution of 2 cm^−1^.

### 4.8. Determination of the Conditions for the Modification of Beech Sawdust

**Figure 11:**
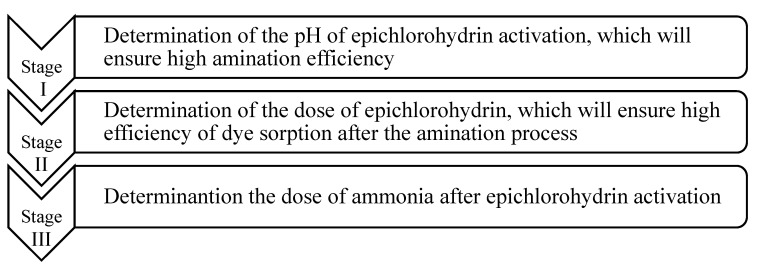
All stages of research into the modification of beech sawdust.

The diagram (Figure 11) shows all the stages of research into the modification of beech sawdust described in Section 4.8.1, Section 4.8.2, Section 4.8.3, Section 4.8.4, Section 4.8.5 and Section 4.8.6.

#### 4.8.1. Determination of the pH of Sawdust Activation with Epichlorohydrin

The first step was to determine the pH value of epichlorohydrin activation at which the sorption process is most effective. The amount of 5 g of beech sawdust was weighed into each of the 18 Erlenmeyer flasks with a capacity of 300 cm^3^ after reaction with sulfuric acid and sodium hydroxide (S-AB) and then 200 cm^3^ of epichlorohydrin solution with pH values of 2.0; 4.0; 6.0; 8.0; 10.0 and 12.0 were added. The doses of activating agent in the solution were chosen so that the ratio of epichlorohydrin functional groups to the number of hydroxyl groups of the sawdust was 1:3, 1:1 and 3:1 (dose 0.417; 1.250; 3.750 g ECH/g S-AB sorbent). The flasks were then left on a shaker with a water bath at 60 °C for 24 h. After the specified time, the sawdust (S-AB-E) was washed with distilled water to get rid of the unreacted activating agent.

The sorption efficiency of the selected anionic (RB5) and cationic (BV10) dyes was then tested on the sorbents prepared in this way at pH values of 3.0; 5.0; 7.0; 9.0 and 11.0. The test time was 2 h

#### 4.8.2. Determining the Dose of the Activating Factor (Epichlorohydrin)

To determine the dose of epichlorohydrin, 2.5 g S-AB d.m. was weighed into 10 Erlenmeyer flasks with a capacity of 300 cm^3^ and 200 cm^3^ of epichlorohydrin solution was then added, the pH being determined using the methodology in Section 4.8.1. The sorbents were prepared according to the methodology in Section 4.4. The doses of epichlorohydrin used in the studies are listed in Table 4.

Each of the prepared sorbents was weighed into conical flasks with a volume of 200 cm^3^ in an amount of 0.5 g d.m. Then, 100 cm^3^ of RB5 dye solution at a concentration of 100 mg/dm^3^ or BV10 dye at a concentration of 10 mg/dm^3^ and a pH of 3.0; 5.0; 7.0; 9.0; 11.0 was added to the flasks. The flasks were then placed on a shaker (150 rpm) for 2 h. Samples of 10 cm^3^ were then obtained to determine the concentration of dye remaining in the solution. The test time was 2 h and pH activation 12.

#### 4.8.3. Determining the Ammonia Dose

To determine the dose of ammonia, 2.5 g of dry matter was weighed into 12 Erlenmeyer flasks with a capacity of 300 cm^3^. S-AB and then 200 cm^3^ of ammonia solution was added. The sorbents were prepared according to the methodology in Section 4.4. The ammonia doses used in the studies are listed in Table 5.

Then, each of the prepared sorbents was weighed into conical flasks with a volume of 200 cm^3^ in the amount of 0.5 g dry matter. The 100 cm^3^ of RB5 dye solution at a concentration of 100 mg/dm^3^ or BV10 at a concentration of 10 mg/dm^3^ was added to the flasks at pH values of 3.0; 5.0; 7.0; 9.0 and 11.0. The flasks were placed on a shaker (150 rpm) for 2 h. Samples (10 cm^3^) were then obtained to determine the concentration of dye remaining in the solution. The test time was 2 h.

#### 4.8.4. Determination of the pH of Epichlorohydrin Activation, Which Will Ensure High Efficiency of Dye Sorption on Aminated Sawdust

To determine the pH of the activation process, 5.0 g of S-AB d.m. was weighed into 18 Erlenmeyer flasks with a capacity of 300 cm^3^ and then 200 cm^3^ of epichlorohydrin solution with pH values of 2.0; 4.0; 6.0; 8.0; 10.0 and 12.0 was added. The doses of activating agent in the solution were chosen so that the ratio of the functional groups of the epichlorohydrin to the number of hydroxyl groups of the sawdust was 1:3, 1:1 and 3:1. The flasks were then left to stand for 24 h on a shaker (150 rpm) with a water bath at 60 °C. After the specified time, the sawdust (S-AB-E) was washed with distilled water to eliminate the unreacted activating agent. Thus, the prepared sorbent was then subjected to the amination process. The doses of 25% aqueous ammonia solution in solution were chosen so that the ratio of amino groups to the number of hydroxyl groups in the sawdust was 1:1 and 10:1. In 36 Erlenmeyer flasks with a capacity of 300 cm^3^, 2.5 g d.m. S-AB-E and then 200 cm^3^ of ammonia solution was added. After the specified time, the sawdust was washed with distilled water to eliminate the unreacted aminating agent.

The sorbents prepared in this way were then used to carry out tests on the effectiveness of the sorption of anionic dye (RB5) and cationic dye (BV10) at pH 3.0; 5.0; 7.0; 9.0 and 11.0. The time of the test was 2 h.

#### 4.8.5. Determination of the Dose of Epichlorohydrin, Which Will Ensure High Efficiency of Dye Sorption on Aminated Sawdust

After determining the pH value of the activation process, the dose of epichlorohydrin at which the amination process was most effective was determined. For this purpose, 5 g of S-AB dry mass was weighed into each of 10 Erlenmeyer flasks with a capacity of 300 cm^3^ and then 200 cm^3^ of epichlorohydrin solution with a pH of 12, as determined in Section 4.8.1, was added. The sorbents were prepared according to the methodology in Section 4. The ratio epichlorohydrin functional groups to the amount of hydroxyl groups of sawdust was 1:10/1:5/1:3/1:2/1:1/2:1/3:1/5:1/8:1/10:1. The doses of epichlorohydrin used in the studies are listed in Section 4.8.4. After reaction with epichlorohydrin, the sorbents were aminated according to the methodology in Section 4.5. The ratio of amino groups to the number of hydroxyl groups of the sawdust was 1:1 and 10:1. For this purpose, 2.5 g d.m. S-AB-E was weighed into 20 Erlenmeyer flasks with a capacity of 300 cm^3^ and then 200 cm^3^ of tab aqueous solution ammonia solution was added. The sorbents were prepared according to the methodology presented in Section 4.4.

The prepared sorbents were then used to perform tests on the sorption efficiency of anionic dye (RB5) and cationic dye (BV10) at pH 3.0; 5.0; 7.0; 9.0 and 11.0.

#### 4.8.6. Determining the Dose of Ammonia After Epichlorohydrin Activation

After determining the pH of the epichlorohydrin activation process and the dose of epichlorohydrin at which the amination process took place, the most effective dose of ammonia was determined. The ratio of epichlorohydrin functional groups to the amount of hydroxyl groups of sawdust was 5:1. The 2.5 g of dry matter of sawdust, which had previously been bathed in an epichlorohydrin solution (S-AB-E) with a pH of 12 and the dose specified in Section 4.8.5, was weighed into 12 Erlenmeyer flasks with a capacity of 300 cm^3^ and then 200 cm^3^ of ammonia solution was added. The doses of 25% aqueous ammonia solution used in the studies are listed in Section 4.8.3. After 24 h, the sawdust (S-AB-E-A) was washed with distilled water to remove the unreacted amine agent.

Thus, the prepared sorbents were then used to perform anionic dye (RB5) sorption efficiency tests at pH 3.0; 5.0; 7.0; 9.0 and 11.0. A batch for cationic dye (BV10) was not carried out, as it was found based on previous studies that any subsequent modification reduces the efficiency of its removal. The time of the test was 2 h and 24 h.

### 4.9. Analytical Methods

#### Determination of Dye Concentration in Solutions

The concentration of dye remaining in the aqueous solution was determined by the spectrophotometric method using the Genesys 20 spectrophotometer from Thermo Scientific (USA). Standard curves were generated for each of the dyes tested. The wavelengths at which the concentration of the remaining dye in the solution were measured and were 600 nm for Reactive Black 5 (RB5) and 554 nm for Basic Violet 10 (BV10).

## 5. Conclusions

In the present study, an increase in the sorption capacity of S-AB sorbent compared to sawdust was observed for both cationic and anionic dyes. The reaction with acid and hydroxide resulted in the removal of interfering substances (i.e., fats, tannins), the exposure of the functional groups responsible for sorption and the loosening of the sorption structure, which facilitated the penetration of the sorbate. A higher sorption capacity was determined for S-AB-A-E in relation to S-AB-A compared to anionic dyes. The removal efficiency of the anionic dyes was greater, as the number of epoxy groups that reacted with the reactive groups of the anionic dyes increased with increasing doses of epichlorohydrin in the activation reaction at pH 12 in the sorbent. The reduction in the efficiency of sorption of cationic pigments, on the other hand, was related to the fact that epichlorohydrin reacts with the –OH groups of the polysaccharides responsible for the sorption of this type of pigment. The modification proposed in the work, which consists in the amination of sawdust in direct reaction with ammonia (S-AB-A), also only increased the sorption efficiency of anionic dyes. Thanks to this reaction, the positive charge on the surface of the sorbent was increased by the introduction of –NH_2_ groups, which increased the electrostatic attraction between the sorbent and the anionic dye and decreased the interaction between the sorbent and the cationic dye. In our own research, the highest sorption capacity for S-AB-E-A was obtained for both anionic dyes. The higher efficiency of removal of anionic dyes by S-AB-E-A than by S-AB-A was related to the larger number of amino groups incorporated into the sorbent structure. The higher efficiency of amination was due to the fact that the reaction with epichlorohydrin yielded the epoxide groups of the sorbent, which are more reactive than the hydroxyl groups of the polysaccharides. The efficiency of amination, i.e., the number of amine functional groups introduced into the structure of the sawdust, depends to a large extent on the conditions of modification.

In our own studies, the highest efficiency of the sorption of anionic dyes on aminated sawdust was achieved after their prior activation with epichlorohydrin at a pH of 12. In an alkaline environment (pH 12), epichlorohydrin reacts with the polysaccharides contained in the structure of the sawdust, giving the sorbent epoxy groups, which then undergo a condensation reaction with the aminating agent. As a result, the sorbent obtains additional amino groups. The effectiveness of the amination also depends on the dosage of the activating and aminating agents. The lowest dose of epichlorohydrin, which enabled effective amination of sawdust, was 6.25 g ECH/g sorbent (ratio 5:1), while the dose of ammonia, which guaranteed high efficiency of the sorption of anionic pigments, was 2.3 g NH_3_/g sorbent (ratio 10:1). When using a modification consisting in the direct reaction of ammonia with sawdust (S-AB-A), a higher ammonia dose of 3.45 g NH_3_/g sorbent (ratio 15:1) is required. Further increases in the dose had no effect on the efficiency of the sorption of anionic dyes.

## Figures and Tables

**Table 1:** Characteristics of beech sawdust.

Component	Dry Matter Content
Cellulose	41.0%
Hemicellulose	27.9%
Lignin	26.7%
Ash	0.1%
Extracts and other ingredients	4.3%

**Table 2:** Characteristics of the dyes used in this study.

Dye Name	Reactive Black 5—(RB5)	Basic Violet 10—(BV10)
Structural formula	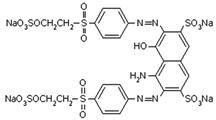	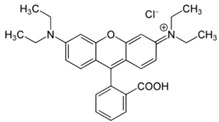
Molar weight	991 g/mol	479 g/mol
λ_max_	600 [nm]	554 [nm]
Type of dye	Anionic—reactive	Cationic
Use	Dyeing of wool, cotton, viscose, polyamide fibers	Dyeing paper, leather, cotton, paint production

**Table 3:** Activating agents used in the study.

Cross-Linking/Activating Agent Name	Epichlorohydrin	25% Ammonia Aqueous Solution
Molecular formula	C_3_H_5_ClO	NH_3_ · H_2_O
Molar mass	93.5 g/mol	35 g/mol
Function groups	1 epoxy group, 1 chloromethyl group	1 amino group
Use	Used in the production of epoxy resins, synthetic glycerin, ethers, waterproof paper, surfactants, insecticides, bactericides and fungicides; solvent for resins and paints	It is used in the tanning and glass industries and in the production of fertilizers, explosives and dyes.

**Table 4:** Activating agent doses.

Ratio of Epichlorohydrin Functional Groups to the Amount of Hydroxyl Groups of Sawdust		Epichlorohydrin Dosage per 1 g s.m. S-AB [g]
1:10	0.1	0.125
1:5	0.2	0.25
1:3	0.3	0.417
1:2	0.5	0.625
1:1	1.0	1.25
2:1	2.0	2.5
3:1	3.0	3.75
5:1	5.0	6.25
8:1	8.0	10.0
10:1	10.0	12.5

**Table 5:** Parameters of the activating agent dose determination.

Ratio of Amino Groups to the Amount of Hydroxyl Groups of Sawdust		Dose of 25% Aqueous Ammonia Solution per 1 g d.m. S-AB [g]
1:10	0.1	0.023
1:5	0.2	0.046
1:3	0.3	0.077
1:2	0.5	0.115
1:1	1.0	0.23
2:1	2.0	0.46
3:1	3.0	0.69
5:1	5.0	1.15
10:1	10.0	2.3
15:1	15.0	3.45
20:1	20.0	4.6
30:1	30.0	6.9

## Data Availability

The data presented in this study are available on request from the corresponding author.
